# *Data on* A parametric temperature dependent potential for β-PbF_2_: A numerical investigation by molecular dynamics

**DOI:** 10.1016/j.dib.2019.104865

**Published:** 2019-11-21

**Authors:** Jeison D. López, Griselda García, Hernando Correa, Edgar Mosquera, Jesús E. Diosa

**Affiliations:** a Departamento de Física, Universidad del Valle, A.A. 25360, Cali, Colombia; b Facultad de Física, Pontificia Universidad Católica de Chile, Casille 306, Santiago, Chile; c Laboratorio de Optoelectrónica, Universidad del Quindío, A.A 2639, Armenia, Colombia; d Centro de Excelencia en Nuevos Materiales (CENM), Universidad del Valle, A.A. 25360, Cali, Colombia

**Keywords:** Ionics conductors, Molecular dynamics, Lead fluoride

## Abstract

This article presents the data on a parametric temperature dependent potential for β-PbF_2_ using molecular dynamics (MD) simulations in the rigid ion approach. The β-PbF_2_ is an important ionic conductor that exhibit a super ionic behavior at 711 K. The understanding of the temperature effect in its properties is crucial for possible applications in electrode for solid state batteries, Cherenkov detectors, and rare earth host for scintillation screen. The simulations were done in the DL_POLY Classic 1.9 package employing the Buckingham pair-potential type. The data have not been reported nor discussed in the research paper to be submitting.

**Table:** Specifications Table

Subject area	*Physics, Materials*
More specific subject area	*Molecular Dynamics Simulation*
Type of data	*Tables (1-4), figures (2)*
How data was acquired	*Molecular Dynamics Simulations*
Data format	*Analyzed*
Experimental factors	*Temperature, fitting potential parameters, Lattice parameter*
Experimental features	*Very brief experimental description*
Data source location	*GTF, Physics Department, Universidad del Valle, Cali, Colombia.*
Data accessibility	*Data are available within this article*
Related research article	*This data article is a direct submission to data in brief having as reference the works of Monteil* et al. [1] *and Silva* et al. [2] *on MD calculation of PbF*_*2*_*, and the Nagornov and Katz* [3] *work about a novel way that include the temperature in the MD potentials.*

**Table:** 

**Value of the Data** • We propose a modification to the potential of β-PbF_2_ proposed by Walker et al. [11], based on the dependence of lattice parameter with temperature. • The calculated data by MD shown a high match with simulated data on structural properties. • The data could be useful to propose different mathematical fitting of temperature-dependent potential for β-PbF_2_

## 1 Data

Most of the bulk properties of fluorite structure ionic crystals, such as CaF_2_ [4], BeF_2_ [5], UO_2_ [3], and PbF_2_ [6] can be simulated by MD using a Buckingham potential type:

(1) $$U\left(r_{ij}\right)=A_{ij}exp\left(\frac{-r_{ij}}{\rho_{ij}}\right)-\frac{C_{ij}}{r_{ij}^{6}}+\frac{q_{i}q_{j}}{r_{ij}}$$

where the first term of the right side is known as the energy repulsion, and represent the electronic overlap, while the second is a well-known dispersion term present in the Lennard-Jones (6–12) potential, which is due to the Coulomb interaction.

In order to modify the β-PbF_2_ potential given by eq. (1)., the *ρ*_*ij*_ coefficient is replaced by a temperature function *ρ*_*ij*_ (*T*), with i=Pb (lead) and j=F (fluorine), respectively. The objective is to find the best *ρ*_*ij*_ (*T*) function, that match well with the experimental lattice parameter data reported in Ref. [7]. Therefore, *ρ*_*ij*_ (*T*) values are presented. All data are shown in Table 1, Table 2. In both tables, the first column are the density values choose in the range from 0.490 to 0.520 eV for the Buckingham fitting parameter, while the second column are computed data of the lattice parameter, *a*_*o*_, obtained using the computer simulation technique of MD.

**Table 1:** Lattice parameter for different *ρ*_*ij*_ values considering the temperature as a parameter.

*ρ*(*T*) (eV)	Lattice parameter *a*_*0*_ (Å) for different temperatures													
	*300 K*	*400 K*	*500 K*	*600 K*	*700 K*	*720 K*	*740 K*	*760 K*	*780 K*	*800 K*	*820 K*	*840 K*	*875 K*	*900 K*
0.490	–	–	–	–	5.716	5.755	5.735	5.759	–	–	–	–	5.903	–
0.495	–	–	–	–	5.819	5.825	5.833	5.840	5.854	5.860	5.868	5.874	5.967	5.912
0.500	5.798	5.823	5.853	5.878	5.920	5.930	5.939	5.944	5.954	5.967	5.976	5.978	6.003	6.025
0.505	5.894	5.911	5.969	5.981	6.030	6.036	6.050	6.054	6.066	6.076	6.088	6.103	6.058	6.159
0.510	5.992	6.016	6.053	6.088	6.136	6.147	6.154	6.166	6.177	6.191	6.199	6.221	6.107	6.107
0.516	6.110	6.142	6.181	6.211	–	–	–	–	–	–	–	–	6.121	–
0.520	6.190	6.204	6.256	6.296	–	–	–	–	–	–	–	–	–	–

**Table 2:** Lattice parameter for different *ρ*_*ij*_ values at 930 K.

*ρ*(*T*) (eV)	Lattice parameter *a*_*0*_ (Å)
0.495	5.941
0.496	5.950
0.497	5.985
0.498	6.002
0.499	6.165
0.500	6.120

## 2 Experimental design, materials, and methods

### 2.1 MD simulation detail

The data reported here was obtained using DL_POLY Classic 1.9 package develop by Smith et al. [8] at the Daresbury Laboratory. In this work, the calculations were performed in a cubic simulation box with 768 atoms and long size of 23.720 Å. VESTA [9] was used to prepare the unit cell, while the supercell was created with Atomsk package [10]. Periodic boundary has been used in order to reproduce the bulk properties. The system was previously equilibrated at environmental conditions: 300 K and 1 atm, respectively. In order to compute the crystal expansion (lattice parameters), the simulations were performed in a NVT ensemble and then relaxed into a NPT ensemble, where the number of the ions (N), temperature (T) and pressure (P) are kept constant. A 5 fs integration time is used to find the ρ(T), then a 1 fs integration time is used to performance a new simulation at the ρ(T) correct values, with finality to corroborate the accurate lattice parameter at each temperature. In both cases, a 10 Å cutoff is employed, and the Ewald sum is used to compute the Coulomb long range potential. The used potential parameters are summarized in Table 3. The data is obtained from Walker et al. [11] as well the modifications proposed in the Section 1.

**Table 3:** Adjustment constants of the potentials that describe the β-PbF_2_ by MD [11].

Atomic pairs	*A*_*ij*_(eV)	$\rho_{ij}$ (Å)	*C*_*ij*_(eV Å^6^)
Pb – Pb	0.0	0.0	0.0
Pb – F	122.7	Tab. 1–2.	0.0
F – F	10255	0.225	107.3

### 2.2 Parametric temperature dependent potential for β-PbF_2_

**Fig. 1:**
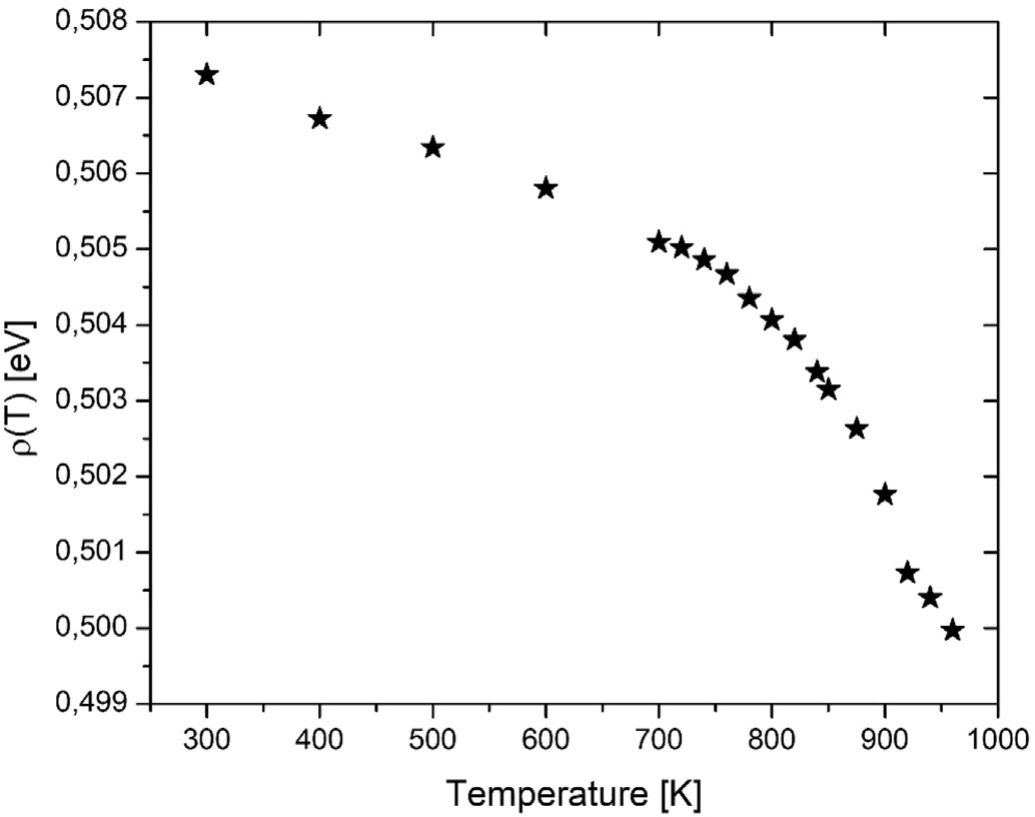
Values for the adjustment parameter *ρ(T) as a function of* temperature.

From the Table 1, Table 2 a linear fitting is done for each temperature. In order to find the better lattice parameter value, thermal expansion measurements for PbF_2_ obtained by Goff et al. [7] by neutron diffraction at different temperatures were employed. The ρ(T) values are shown in Fig. 1.

In order to make a first approximation on the validation of the potential data shown in Fig. 1., the enthalpy of the atomic system was recorded (refer to Table 4).

**Table 4:** Values of the enthalpy as a function of temperature for β-PbF_2_.

Temperature (K)	Enthalpy (kJ mol^−1^)
300	−2239.19618
350	−2236.83185
370	−2235.87824
400	−2234.44629
420	−2233.46642
450	−2231.99567
470	−2230.98597
500	−2229.42186
520	−2228.37485
550	−2226.68132
570	−2225.5548
600	−2223.77367
620	−2222.5076
650	−2220.55895
670	−2219.266
700	−2217.2881
720	−2215.84356
750	−2213.80256
770	−2212.31626
800	−2210.2459

**Fig. 2:**
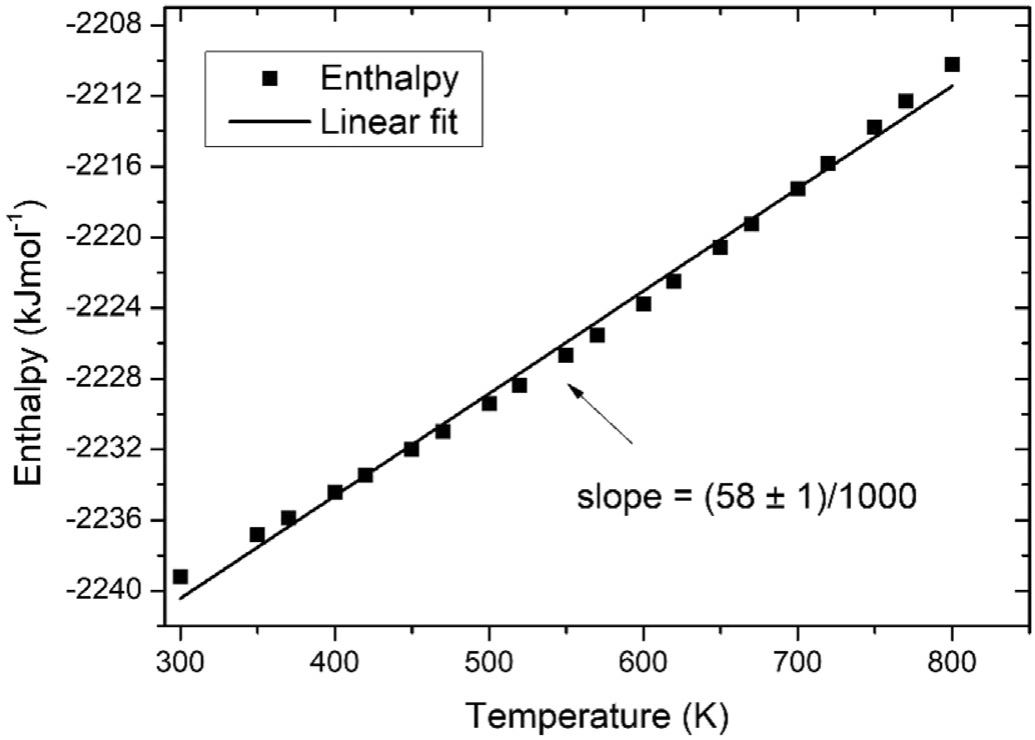
The enthalpy for β-PbF_2_. In filled square, data obtained by MD; in solid line, the linear fit.

The specific heat capacity at constant pressure, *C*_*p*_, is calculated from Table 4 and the slope of the linear fit shown in Fig. 2. The *C*_*p*_ value obtained by MD is 58 ± 1 J mol^−1^ K^−1^ which is in acceptable agreement with reported value of 69 ± 7 J mol^−1^ K^−1^ between 400 and 640 K [12].
